# Draft Genome Sequences of Two Thermotolerant Cyanobacterial Strains Isolated from Hot Springs

**DOI:** 10.1128/genomeA.01548-17

**Published:** 2018-02-01

**Authors:** Kirill S. Mironov, Maria A. Sinetova, Elena V. Kupriyanova, Vera V. Ustinova, Anna Y. Kozlova, Ekaterina M. Messineva, David A. Gabrielyan, Vladimir S. Bedbenov, Bolatkhan K. Zayadan, Dmitry A. Los

**Affiliations:** a Institute of Plant Physiology, Russian Academy of Sciences, Moscow, Russia; b Microbiology Department, Central Tuberculosis Research Institute, Moscow, Russia; c Department of Biotechnology, Faculty of Biology and Biotechnology, Al-Farabi Kazakh National University, Almaty, Kazakhstan

## Abstract

We report here two draft cyanobacterial genome sequences, those of *Cyanobacterium aponinum* IPPAS B-1201, isolated from a hot spring in the Turgen Gorge (Kazakhstan), and the uncharacterized cyanobacterium IPPAS B*-*1203, isolated from a hot spring in Karlovy Vary (Czech Republic). These two strains were deposited at the Collection of Microalgae (IPPAS) of the Timiryazev Institute of Plant Physiology.

## GENOME ANNOUNCEMENT

*Cyanobacterium aponinum* strain IPPAS B-1201 was isolated from an environmental sample harvested at a Turgen Gorge (Republic of Kazakhstan) hot spring with an average temperature of 45°C. The second cyanobacterial strain, IPPAS B-1203, was isolated from an environmental sample harvested at a Karlovy Vary (Czech Republic) hot spring with an average temperature of 40 to 50°C. Both cultures were purified from bacterial contaminants and cultivated as axenic cyanobacterial strains in BG-11 medium. The cell cultures were deposited in the Collection of Microalgae (IPPAS) of the Timiryazev Institute of Plant Physiology, Moscow, Russia.

Cyanobacterial cells were lysed by incubation with saturated iodide solution, followed by lysozyme treatment and lysis in 4% SDS at 75°C (1). Lysate was treated with a phenol-chloroform mixture for DNA purification. Genomic DNA was finally precipitated with ethanol and pelleted. DNA shearing and library preparation for sequencing were carried out using the NEBNext Fast DNA library prep set for Ion Torrent. A library size selection of 490-bp fragments was conducted via agarose gel electrophoresis. Ion Sphere Particles were generated in the Ion OneTouch 2 system. Sequencing was performed on Ion PGM with Hi-Q View chemistry in 400-bp format on an Ion 316 Chip v2. For MiSeq sequencing, a library was prepared using the Nextera XT DNA library prep kit. The MiSeq run was performed in a 600-bp paired-end format.

The genomes of both strains were assembled using SPAdes 3.11.1 (2). The qualities of the assemblies were analyzed with QUAST (3). For the draft genome of IPPAS B-1201, the median coverage was approximately 220×, and the *N*_50_ value was 93,055 bp. The approximate genome size is 4.3 Mb, and the average G+C content was estimated to be 34.9%.

For a hybrid assembly of the IPPAS B-1203 genome, reads from Ion PGM and MiSeq were used (the first draft was assembled with Ion Torrent reads, and the secondary assembly was performed on Illumina reads with contigs from the Ion Torrent assembly with the parameter “--trusted-contigs”). The median coverage was approximately 30×, and the *N*_50_ value was 398,834 bp. The number of contigs was 57. The approximate genome size is 5.9 Mb, and the average G+C content was estimated to be 41.7%.

Genomes were annotated using automated NCBI PGAP. The IPPAS B-1201 genome contained 3,649 genes in total, with 3,506 genes coding for proteins, 97 pseudogenes, 3 rRNA-coding sequences (5S, 16S, and 23S rRNAs), 39 tRNAs, and 4 noncoding RNAs. Eight clustered regularly interspaced short palindromic repeat (CRISPR) arrays have been found in the genome.

The IPPAS B-1203 genome contained 5,374 genes in total, with 5,072 genes coding for proteins, 256 pseudogenes, 3 rRNA-coding sequences (5S, 16S, and 23S rRNAs), 39 tRNAs, and 4 noncoding RNAs. Two CRISPR arrays were found in the genome. The strain belongs to a taxonomically uncharacterized group of a recently proposed order, *Chroococcidiopsidales* (4). The most closely related (by identity) genera of cyanobacteria from reference representative genomes are *Gloeocapsopsis* and *Gloeocapsa*.

### Accession number(s).

The genome sequence of IPPAS B-1201 has been deposited in GenBank under BioProject number PRJNA415147, BioSample number SAMN07816679, assembly ASM273600v1, accession number PEBC00000000, and SRA accession number SRR6208854. The genome sequence of IPPAS B-1203 has been deposited in GenBank under BioProject number PRJNA415142, BioSample number SAMN07816539, assembly ASM274997v1, accession number PEIG00000000, and SRA accession numbers SRX3340725 and SRX3340726.
